# Editorial: Pain education research: advances, innovations, and challenges

**DOI:** 10.3389/fpain.2024.1403461

**Published:** 2024-04-08

**Authors:** Mark I. Johnson, Ylisabyth S. Bradshaw, Scott M. Fishman, Kate Thompson, Judy Watt-Watson

**Affiliations:** ^1^ Centre for Pain Research, School of Health, Leeds Beckett University, Leeds, United Kingdom; ^2^ Department of Public Health and Community Medicine, Tufts University School of Medicine, Boston, MA, United States; ^3^ Division of Pain Medicine, Davis School of Medicine, University of California, Sacramento, CA, United States; ^4^ L. S. Bloomberg Faculty of Nursing, University of Toronto, Toronto, ON, Canada

**Keywords:** pain education, pain education competencies, pain education curricula, translational research, healthcare professionals, public pain education, pain neuroscience education, pain psychoeducation

**Editorial on the Research Topic** Pain education research: advances, innovations, and challenges

## Introduction

Worldwide consensus has identified pain education for health care professionals as inadequate to meet the clinical need for pain assessment and management. In the past decade, several professional organizations and academic institutions have taken this challenge and formulated curricula and training (often interprofessional) informed by the neuroscience of education and learning. As the cumulative experience among pain educators worldwide grows and evolves, a body of work has developed that systematically assesses the efficacy and real-world effectiveness of diverse approaches to pain education as a crucial stage in the translation of basic and applied knowledge into clinical practice.

Challenges facing pain educators include deciding upon and conveying the content to be transmitted, how best to use competencies related to this content, and how to tailor general educational approaches to best meet the needs of health care professionals dealing with patients with pain. Other issues include engaging patients and the public, including policy makers, in shared efforts to reduce unnecessary pain and suffering and optimize clinical outcomes associated with the entire spectrum of painful experiences.

This Research Topic provides a collection of articles addressing previous and ongoing barriers to the education of health care professionals, including competencies, and evidence-based approaches to overcoming these barriers, complemented by concurrent advances in patient and public education. We invited contributions on, but not limited to, the following topics:

• Content of pain education for health care professionals and its refinement according to changes in scientific knowledge and clinical practice.
• Methodologies synthesizing and translating pain-related content to ensure application to clinical care of people with pain across the lifespan.
• Frameworks and approaches to overcome challenges encountered by educators trying to facilitate learning about contemporary topics.
• Gaps in post-graduate and continuing education for licensed health professionals.

Eleven articles were accepted for inclusion in the Research Topic.

## Overview of contributions

Contributions to the Research Topic provide a systematic evaluation of pain education content and methods, and novel perspectives and future directions for both practice and research. The collection includes the development and evaluation of various frameworks and approaches to overcome challenges encountered by educators trying to facilitate learning about contemporary approaches to assess and manage complex pain presentations, within the broader context of health care system change.

In the first of two contributions, Mardian et al. draw attention to the need for transformation in pain education and culture to improve clinical practice, through a lens of “didactic dissonance”—a disconnect between what is taught in classroom settings and what learners observe in clinical settings. They propose a process based on transformative learning theory to assist learners in exploring factors that create and perpetuate the education-practice disconnect, opening avenues of exploration for transformation in both educational and clinical practice. The findings of a qualitative study by Thompson et al. support this viewpoint by providing evidence that stakeholders of physiotherapy education emphasise the importance of preparing graduates for the challenges faced when encountering “real” people from diverse sociocultural backgrounds. Thompson et al. suggest that pain education in health care can be improved through a curriculum of practically engaging pain scenario's reflecting the challenges faced in clinical practice.

The contribution by Siaton et al. demonstrates the challenges faced by educators and practitioners in the management of complex presentations, particularly related to older people with pain comorbidities. Siaton et al. report the use of a mixed methods approach to develop and pilot a Pain in Aging, Educational Assessment of Need (PAEAN) instrument to inform pain, comorbidities, and clinical decision-making associated with the complexities of assessing and managing geriatric pain. Their findings suggest that several factors impact pain-related clinical decision-making and that it is feasible to survey healthcare practitioners about the influence of comorbidities on decisions in the care of older adults with pain. The contribution by Soenarto et al. provides evidence that mnemonics are a helpful tool to develop knowledge of and skills for assessing pain in clinical consultation during a simulation-based educational workshop. Soenarto et al. found that medical students' knowledge and skills of assessing chronic pain was improved using the PQRST (P, provoke and palliate; Q, quality; R, region and radiation; S, severity; T, time) mnemonic, although the addition of an ACT-UP (A, activity; C, coping; T, think; U, upset; P, people) was no better than using PQSRT alone. Soenarto et al. conclude that mnemonics are useful and can be integrated into various learning contexts such as lectures, demonstrations, simulations, and interactions with patients.

The contribution by Shipton et al. provides a useful approach to the complex process of curriculum change and supports the need for more formalised procedures to design, develop and evaluate the pain medicine curriculum. Shipton et al. describe how they conceptualised and developed a purposeful method to facilitate structured integration of pain education into the medical curriculum. Their Pain Medicine Curriculum Framework comprises future healthcare practice needs, the competencies and capabilities required of graduates, the teaching, learning and assessment methods to use, and institutional parameters. The contribution of Cao and Van Deusen provides evidence for the integration of opioid use disorder and chronic pain content within medical curricula. Their topic review revealed a lack of emphasis on chronic pain education, biopsychosocial approaches, and interprofessional learning in current US medical school curricula; and their evaluation of twelve winning student-designed opioid use disorder curricula utilised more diverse learning activities and assessment methods than current US medical school curricula.

In their additional contribution, Mardian et al. describe the “hidden curriculum” as a vehicle by which students learn values, attitudes, beliefs, and related behaviors important to medicine, and that this “hidden curriculum” is entrenched in a biomedical model of practice. Mardian et al. explain how they employed the Implicit Bias Recognition and Management tool to “flip” this hidden biomedical curriculum towards a sociopsychobiological model of care. Similarly, the contribution by Ng et al. reports the application of the behavioural change wheel to guide the implementation of a biopsychosocial approach to musculoskeletal pain care; they propose a worked example on how to operationalise the framework. In their contribution, Darnall et al. argue a need to overcome system-level barriers associated with the biomedically-dominant culture that marginalises education about, and access to, high-quality, evidenced-based behavioural pain treatments for youth and adults. Darnall et al. review literature to reveal several innovative digital treatment formats, technologies, and clinician trainings and offer clinical recommendations and future directions for research. The examples of evidence-informed strategies to assess, identify and analyse biopsychosocial factors provided in these contributions can be used by healthcare professionals and educationalists to strengthen a whole-of-system adoption of a biopsychosocial approach to pain care.

Technological advances have driven a shift towards online learning, and the contribution by Dao and Cao provides evidence to support the utility of this educational medium. They found that improvements in physiological knowledge and ability to work together in interprofessional teams achieved during in-person training using the Supervised Student Inter-professional Pain Clinic Program (SSIPCP) were maintained when the program had to be delivered online using Zoom due to the COVID-19 pandemic. However, students using Zoom expressed a preference for in-person activities. The contribution by Lalloo et al. describes how they integrated and evaluated pediatric-pain core competency education within the Extension for Community Healthcare Outcomes (ECHO®) for Pain model. The ECHO® model delivers online education to interprofessional healthcare providers through virtual clinics to cultivate a community of practice, and Lalloo et al. provide evidence that the Pediatric Project ECHO® for Pain improved knowledge and self-efficacy in learners and had high usability with clinically realistic cases.

## Impact of contributions

Contributions to this Research Topic address advances, innovations, and challenges in pain education research and provide evidence of a need to safeguard adequate and appropriate coverage of pain education in health professional curricula. In doing so, educationists are tasked with designing learning situations that align with contemporary pain knowledge, including sociopsychological aspects of pain and its management within the biopsychosocial model of care. Through providing examples of frameworks that are being used and evaluated to assist curriculum development, this eBook offers perspectives on how the challenges of reconceptualising pain education can be overcome. In addition to the focus on students, approaches address education for the educator who is developing and implementing curriculum, in both uni- and/or interprofessional settings. Contributions discuss the need to formalise approaches to design, develop, and evaluate pain curricula, and to provide learning opportunities that progress knowledge and skills on psychosocial aspects of pain. These discussions can be used to inform future directions of research and practice. Of priority is the development and implementation of authentic patient scenarios across the lifespan, co-created with people living with pain, that reflect the complexities and cultural-diversity of real-life clinical practice.

